# Burden of S*higella* spp and *Vibrio* spp, and their antibiotic sensitivity pattern in the patients with acute gastroenteritis in tertiary care hospital in Nepal

**DOI:** 10.1186/s13104-019-4722-1

**Published:** 2019-10-26

**Authors:** Shiv Kumar Sah, Shreejana Basnet, Sushma Shrestha, Kusum Ghale, Sabita Tamang, Dipendra Kumar Mandal, Sher Bahadur Pun

**Affiliations:** 1 Faculty of Pharmaceutical Science, Little Buddha College of Health Science, Purbanchal University, Minbhawan, Kathmandu, Nepal; 2 Sukraraj Tropical and Infectious Disease Hospital, Teku, Nepal; 3 Minbhawan, GPO box-26508, Kathmandu, Nepal

**Keywords:** Clinical profile, Acute gastroenteritis, Bacterial isolates, Resistance pattern

## Abstract

**Objectives:**

The present study aims to investigate the etiology, clinical profile and resistance pattern of the isolated pathogens in Nepalese adults with acute gastroenteritis. This cross-sectional study was conducted at Sukraraj Tropical and Infectious Disease Hospital, from April 2016 to Sep 2017. Subjects’ ages 14 or above, presenting with gastroenteritis with positive stool culture were enrolled for analysis.

**Results:**

Of total 153 patients, 47.72% subjects confirmed the presence of bacterial infection. *Vibrio cholera*e spp and *Shigella* spp were detected in 36.6% and 23.28% respectively. The most common resistance among *Vibrio cholera*e was to nitrofurantoin (92.8%), cotrimoxazole (92.8%) and nalidixic acid (92.8%). Among 17 isolates of *Shigella* spp, the most frequent drug resistant was observed in ampicillin (64.7%), nalidixic acid (58.8%), ceftriaxone (47%). Chloramphenicol (94.1%), tetracycline (88.2%), and cotrimoxazole (82.3%) were found to be the most sensitive towards this pathogen. High rate of diarrhea due to bacterial infection, especially *Shigella* spp and *Vibrio* spp and their high rate of drug resistance emphasize an urgent need of designing a surveillance system for antimicrobial resistance in Nepalese setting.

## Introduction

Diarrhoeal diseases remain a major cause of morbidity and mortality in all age groups in impoverished areas of South East Asia [1–5]. A wide range of bacteria, parasite, virus remained the responsible for the disease [6]. The clinical feature and pattern of isolation varies from place to place, depending on the local climate and geography, and socioeconomic factors [7]. Among the different pathogens responsible for diarrhea, *Shigella* spp. play an important role in causing inflammatory diarrhea and dysentery, and can be considered a global health problem [8–11], causing significant morbidity and mortality in developing countries [8, 9, 12]. Similarly, in Nepal, one of the most important causes of acute diarrhea in children ischolera [13]. Over the past decades, *Shigella* spp and *Vibrio cholera*e have become progressively resistant to the most widely-used and inexpensive antimicrobials [7, 12, 13]. Most of the studies in diarrheal disease globally and in South Asia have focused on children under 5 years old and under rural setting [14–16]. Consequently, only limited information about epidemiology and etiology of diarrheal infections in adults residing in developing countries are available [17–19]. Therefore, the present study aims to describe the etiology, clinical profile and resistance pattern of the isolated pathogens in Nepalese adults with acute diarrhea.

## Main text

### Methods

#### Study design and duration

This was a cross-sectional observational study, conducted over 6 month period from April 2016 to Sep 2017.

Setting: The present study, conducted at Sukraraj Tropical and Infectious Disease Hospital (STIDH), established in 1933, is the only Infectious & Tropical Disease Hospital in Kathmandu, Nepal. This is the national referral hospital with a capacity of 100 beds. It receives patients from all over the country and referred patients from hospitals in Kathmandu Valley.

#### Patient’s selection and data collection

Subjects ages 14 or above, presenting with acute gastroenteritis were sampled during 6 month study period. Demographic including age and sex, clinical features, including body temperature, stool type, abdominal pain, and vomiting and laboratory report were assessed at study entry.

#### Isolation and identification of pathogen

Faecal samples were inoculated on MacConkey agar (Hi-media) plates. The feces samples were cultured on MacConkey agar, xylose lysine Deosxycholate agar; Thiosulphate citrate bile salt sucrose agar and Selenite F enrichment broth (Oxoid, England) were used for isolation of *Shigella and Vibrio*. Culture negative specimens on primary solid media were sub-cultured from the enrichment broth to primary solid media to improve recovery of the isolates. All inoculated media were incubated at 37 °C for 18–24 h. After overnight incubation, non-lactose fermenters were further identified by biochemical tests using appropriate media namely: triple sugar Iron agar for carbohydrate fermentation test, urea agar for the urea utilization test, sulphide Indole motility test for indole production, Simmon citrate agar for citrate utilization, motility agar for motility test, lysine agar for lysine utilization test (all Oxoid, England). The plates were incubated at 37  °C for a minimum of 24 h. Plates exhibiting no growth were incubated for another 24 h. All isolated colonies growing in the MAC agar were processed further for identification.

Identification of the isolates was done by the standard microbiological techniques which involved morphological appearance of the colonies, Grams staining reactions, catalase test, oxidase test and other biochemical properties.

#### Antimicrobial susceptibility testing

The antimicrobial susceptibility of all identified isolates were done with various antibiotics using Mueller–Hinton agar (MHA) by modified Kirby–Bauer disk diffusion technique following the criteria of Clinical and Laboratory Standards Institute (CLSI) [20]. Different strains were used as quality control like *P. aeruginosa* ATCC 27853, *E. coli* ATCC 25922, *S. aureus* ATCC 25923.

### Statistical analysis

Descriptive data were generated. Continuous data were presented in Mean ± SD, and frequency table were generated for categorical variables. Chi square test and Fisher exact test were performed for comparison of categorical variables. A P value < 0.05 was considered to be statistically significant. All data were entered and managed in SPSS version 20.

### Ethical aspects

The study complies with the standards of the Declaration of Helsinki and has been approved by research committee of Sukraraj Tropical and Infectious Disease Hospital (STIDH). Anonymous forms were used to collect the data to assure confidentiality.

## Results

Demographic characteristics as well as clinical profiles of the study subjects are shown in Table 1. Of total 153 patients, mean ages were 38.89 ± 1.68 years, and more than half 80 (52.3%) of the study population were male. With regards to symptoms, loose motion was recorded in majority 153 (100%) of the subjects, followed by dehydration 150 (98%), abdominal pain 119 (77.8%), vomiting 99 (64.7%), nausea 49 (32%) and burning maturation 24 (15.7%). On examination, 51(33.3%) of the patients were found to be febrile.

**Table 1:** Demographics and clinical characteristics

Characteristics	Inference
N	153
Age, mean ± SD (min–max)	38.89 ± 1.68 (14–88)
Sex, n (%)	
Male	80 (52.3)
Female	73 (47.7)
Symptoms, n (%)	
Dehydration	150 (98.0)
Loose motion	153 (100.0)
Abdominal pain	119 (77.8)
Vomiting	99 (64.7)
Fever	51 (33.3)
Nausea	49 (32.0)
Burning micturition	24 (15.7)
Confirmed enteric infection, n (%)	
Parasite (+), n (%)	40 (26.1)
Bacterial infection	73 (47.71)
Multi drug resistance	56 (36.6)
*Shigella* spp, n (%)	17 (11.1%)
*Shigella flexneri* *Shigella sonnei*	7/17 (41.17) 10/17 (58.82)
*Vibrio* spp, n (%)	56/153 (36.6)

Among the 153 stool samples collected, the estimated prevalence of *Shigella* spp and *Vibrio* spp were 11.1%, and 36.6% respectively. Of total 17 Shigella isolates, *Shigella flexneri* were identified as 41.17% and *Shigella sonnei* as 58.8%.

The drug susceptibility patterns of 56 isolates of *Vibrio cholerae* spp were determined (Table 2). The high resistance rate among *Vibrio cholerae* was to nitrofurantoin 52 (92.8%), cotrimoxazole 52 (92.8%) and nalidixic acid 52 (92.8%), ciprofloxacin 48 (85.7%), ampicillin 26 (46.4%), chloramphenicol 23 (41%). However, 53 (94.6%) strain of *Vibrio* spp were sensitive to tetracycline.

**Table 2:** Drug susceptibility pattern of *Vibrio* spp

Drugs	*Vibrio cholerae* (N = 56)		
	Resistant (%)	Sensitivity (%)	Intermediate (%)
Tetracycline	1 (1.7%)	53 (94.6%)	2 (3.5%)
Ciprofloxacin	48 (85.7%)	5 (8.9%)	3 (5.3%)
Cotrimoxazole	52 (92.8%)	2 (3.5%)	2 (3.5%)
Chloramphenicol	23 (41%)	1 (1.7%)	32 (57.1%)
Nalidixic acid	52 (92.8%)	2 (3.7%)	2 (3.7%)
Nitrofurantoin	52 (92.8%)	0 (0.00)	4 (7.1%)
Azithromicin	12 (21.4%)	19 (33.9%)	25 (44.6%)
Norfloxacin	0 (0.00)	25 (44.6%)	31 (55.3%)
Ampicillin	26 (46.4%)	3 (5.3%)	27 (48.2%)

Among 17 isolates of *Shigella* spp, the resistant rate was high for ampicillin 11 (64.7%), nalidixic acid 10 (58.8%), ceftriaxone 8 (47%) and ciprofloxacin 7 (41.17%).

However, more than 90% strain of these isolates were sensitive to chloramphenicol 16/17 (94.1%), and more than 80% strain of these isolates were sensitive to tetracycline 15/17 (88.2%) and cotrimoxazole 14/17 (82.3%) (Table 3).

**Table 3:** Drug sensitivity pattern of * Shigella * spp

Drugs	*Shigella *spp (N = 17)		
	Resistance (%)	Sensitivity (%)	Intermediate (%)
Tetracycline	2 (11.7%)	15 (88.2%)	0 (0.00)
Ciprofloxacin	7 (41.17%)	9 (52.9%)	1 (5.8%)
Cotrimoxazole	3 (17.6%)	14 (82.3%)	0 (0.00)
Chloramphenicol	1 (5.8%)	16 (94.1%)	0 (0.00)
Nalidixic acid	10 (58.2%)	4 (23.5%)	3 (17.64%)
Norfloxacin	0 (0.00)	3 (17.6%)	14 (82.3%)
Ampicillin	11 (64.7%)	4 (23.5%)	2 (11.7%)
Ofloxacin	0 (0.00)	2 (11.7%)	15 (88.2%)
Ceftriaxone	8 (47%)	6 (35.2%)	3 (17.6%)

## Discussion

*Shigella* and *Vibrio* spp infections are global public health burden, and have been increasingly acquiring resistance to commonly used antibiotics, posing serious threats in antimicrobial treatment worldwide. The burden of *Shigella* spp and *Vibrio* spp in Nepal are mostly documented in children population, however, the data in adult population is lacking.

In the present study at least one enteric pathogen was isolated from 72.52% of patients. Among these, the most frequently reported pathogen was bacterial isolate (47.71%). Parasite was identified notably in higher number of population (26.1%). In comparison, studies from sub-Saharan country demonstrated 47 (60.2%) and 31 (39.7%) parasitic and bacterial infection respectively [21].

In the present study, bacterial infection remained the major etiology of the disease, with estimated prevalence of *Vibrio* spp and *Shigella* spp 36.6% and 11.1% respectively.

Available literatures suggest varying prevalence of *Shigella* spp across the nations, ranges between 5 and 16.8% [22–25]. The prevalence *of Shigella* spp in under 5 years of age in Nepal were found to be 52.2% [26]. This discrepancy may be due to the differences in sample size, variation in the age group, level of public health, status of safe drinking water among the population, geographical distribution, seasonal variation of the isolates at which the sample were collected, and personal hygiene of the target population.

In the present study, *Shigella sonnei* 10 (58.8%) remained the most common species followed by *Shigella flexneri* 7 (41.17%), which is in consistent with the results of the study in pediatric population from Teharan n Farahani et al., where 60.8% reported to be *Shigella sonnei* and 39.2% *S. flexeneri* [27]. A similar results were shown by Telebreza et al. [28] in pediatric population; 61.1% *Shigella sonnei,* followed by *S. flexneri* (27.8%). However, in pediatric population in Nepal a low prevalence of *Shigella sonnei* (25%) and *S. flexneri* 20.83% were found [26]. The prevalence of *Shigella flexneri* (7.5%) and *S. sonnei* (5.2%) reported in Karaj, Teharan [11] were shown to be lower than ours.

In a previous study by Karki Ravindra [13], *Vibrio cholerae* was found to be 21.1%. In comparison, our study showed high prevalent of *Vibrio* spp (36.6%). In previous studies, bacterial infection occurred more frequently during rainy season [11, 13, 21]. In consistent with the results of these studies, the proportions of *Shigella* and *Vibrio spp* infection in this study were predominantly appeared to be higher during rainy season (June and July). In Nepal, the rainy season includes the warmest month and the period of heavy rain and floods. Heavy rain and floods are responsible for poor sanitation and increase human contact with waste water, in turn infection. However, more data in subsequent years are needed to elucidate the seasonality of this pathogen in this region.

Available literatures suggest that abdominal pain (72.6%), weakness (41.9%), and vomiting (36.5%) are the most common symptoms in diarrhoeic patients [21]. In our study, loose motion was recorded in majority 153 (100%) of the subjects, followed by dehydration 150 (98%), abdominal pain 119 (77.8%), vomiting 99 (64.7%), nausea 49 (32%) and burning maturation 24 (15.7%). On examination 51 (33.3%) of the patients were found to be febrile. Different type of stool experienced on direct observation were: watery stool 115 (75%), blood mixed stool 4 (2.6%), water + blood mixed + mucous stool in 10 (6.5%) and watery + mucous stool 14 (9.2%). These differences may be defined by the incomparable proportion of bacterial and parasite infection. Viral infection and patient host characteristics in the cited article could be another reason.

In previous study [11], the most common resistance was to tetracycline (73.5%), trimethoprim–sulphamethoxazole (70.4%), and amoxicillin–clavulanic acid (50.0%). Resistance to cefixime, ciprofloxacin, ceftriaxone, and nalidixic acid was observed in 6.1%, 3.1%, 2.0%, and 1.0% of the isolates respectively. In our study, about 37% *Shigella* spp had more than one drug resistant, and among 17 isolates of *Shigella* spp, the most frequent drug resistant was to ampicillin 11 (64.7%), nalidixic acid 10 (58.8%), ceftriaxone 8 (47%), ciprofloxacin 7 (41.17%).

In our study, *Vibrio cholerae* was detected in 56 (36.6%) of the population. Among these isolates, nitrofurantoin 52 (92.8%), cotrimoxazole 52 (92.8%) and nalidixic acid 52 (92.8%), were found to be highly resistant which shows a consistency to the study revealed by Karki Rabindra [13], where Furozolidine, cotrimoxazole and nalidixic acid was found to be 100% resistant towards this pathogen. In a earlier study [13], ciprofloxacin (100%), tetracycline (100%), ampicillin (73.6%) and erythromycin (88.4%) was highly sensitive to this isolate. In our study, ciprofloxacin 48 (85.7%), ampicillin 26 (46.4%), chloramphenicol 23 (41%) were appeared to be resistant. No growth 27 (48.2%) was found in ampicillin, and only 3 (5.3%) was found to be sensitive to ampicillin. The finding indicates that the trend of resistance pattern of *V. cholerae* spp towards ciprofloxacin and ampicillin are on increase, and a proper surveillance measure is necessary so as to prevent this emergence of bacterial resistance. In previous study, tetracycline 53 (94.6%) appeared the most sensitive to this isolate which compiles the results of previous study by Karki [13]. In our study, ofloxacin 25 (44.6%) and azithromycin 19 (33.9%) appeared fairly sensitive to this pathogen. However, a similar study in a large population is necessary to explore the clear picture.

## Conclusion

Our study confirmed a high rate of *Shigella * spp and *Vibrio cholerae* in Nepalese adult population, which appeared as an important aetiological agent of acute diarrhea with high rate of drug resistance. Thus, the results of this study emphasize an urgent need of designing a surveillance system for antimicrobial resistance in Nepal and the introduction of integrated guidelines for the appropriate use of antibiotics.

## Limitation

This study was single centered hospital based study. So, the isolates resulted in this study may not be the representative of the entire country.

## Data Availability

The data sets used during the current study are available from corresponding authors on reasonable request.

## Abbreviations

STIDH: Sukraraj Tropical and Infectious Disease Hospital
SD: standard deviation
Jun: June
Apr: April

## References

[1] Bern C, Martines J, De Zoysa I, Glass R (1992). The magnitude of the global problem of diarrhoeal disease: a ten-year update. Bull World Health Organ.

[2] Agtini MD, Soeharno R, Lesmana M, Punjabi NH, Simanjuntak C, Wangsasaputra F (2005). The burden of diarrhoea, shigellosis, and cholera in North Jakarta, Indonesia: findings from 24 months surveillance. BMC Infect Dis.

[3] Kosek M, Bern C, Guerrant RL (2003). The global burden of diarrhoeal disease, as estimated from studies published between 1992 and 2000. Bull World Health Organ.

[4] Snyder JD, Merson MH (1982). The magnitude of the global problem of acute diarrhoeal disease: a review of active surveillance data. Bull World Health Organ.

[5] Boschi-Pinto C, Velebit L, Shibuya K (2008). Estimating child mortality due to diarrhoea in developing countries. Bull World Health Organ.

[6] Guerrant RL, Hughes JM, Lima NL, Crane J (1990). Diarrhea in developed and developing countries: magnitude, special settings, and etiologies. Rev Infect Dis.

[7] Thapar N, Sanderson IR (2004). Diarrhoea in children: an interface between developing and developed countries. The Lancet..

[8] Georges M, Wachsmuth I, Meunier D, Nebout N, Didier F, Siopathis M (1984). Parasitic, bacterial, and viral enteric pathogens associated with diarrhea in the Central African Republic. J Clin Microbiol.

[9] Rosenberg ML, Weissman JB, Gangarosa EJ, Reller LB, Beasley RP (1976). Shigellosis in the United States: ten-year review of nationwide surveillance, 1964–1973. Am J Epidemiol.

[10] Hossain MA, Albert MJ, Hasan KZ (1990). Epidemiology of shigellosis in Teknaf, a coastal area of Bangladesh: a 10-year survey. Epidemiol Infect.

[11] MoezArdalan K, Zali MR, Dallal MMS, Hemami MR, Salmanzadeh-Ahrabi S (2003). Prevalence and pattern of antimicrobial resistance of *Shigella* species among patients with acute diarrhoea in Karaj, Tehran, Iran. J Health Popul Nutr.

[12] Bennish ML (1991). Potentially lethal complications of shigellosis. Rev Infect Dis.

[13] Karki R, Bhatta DR, Malla S, Dumre SP (2010). Cholera incidence among patients with diarrhea visiting National Public Health Laboratory, Nepal. Jpn J Infect Dis.

[14] Brooks WA, Santosham M, Naheed A, Goswami D, Wahed MA, Diener-West M (2005). Effect of weekly zinc supplements on incidence of pneumonia and diarrhoea in children younger than 2 years in an urban, low-income population in Bangladesh: randomised controlled trial. The Lancet..

[15] Kotloff KL, Nataro JP, Blackwelder WC, Nasrin D, Farag TH, Panchalingam S (2013). Burden and aetiology of diarrhoeal disease in infants and young children in developing countries (the Global Enteric Multicenter Study, GEMS): a prospective, case-control study. The Lancet..

[16] Safa A, Nair GB, Kong RYC (2010). Evolution of new variants of *Vibrio cholerae* O1. Trends Microbiol.

[17] Shapiro RL, Kumar L, Phillips-Howard P, Wells JG, Adcock P, Brooks J (2001). Antimicrobial-resistant bacterial diarrhea in rural western Kenya. J Infect Dis.

[18] Okeke IN, Ojo O, Lamikanra A, Kaper JB (2003). Etiology of acute diarrhea in adults in southwestern Nigeria. J Clin Microbiol.

[19] Sambe-Ba B, Espié E, Faye ME, Timbiné LG, Sembene M, Gassama-Sow A (2013). Community-acquired diarrhea among children and adults in urban settings in Senegal: clinical, epidemiological and microbiological aspects. BMC Infect Dis.

[20] Wikler MA. Performance standards for antimicrobial disk susceptibility tests: approved standard. Clinical and Laboratory Standards Institute. 2006.

[21] Sambe-Ba B, Espié E, Faye ME, Timbiné LG, Sembene M, Gassama-Sow A (2013). Community-acquired diarrhea among children and adults in urban settings in Senegal: clinical, epidemiological and microbiological aspects. BMC Infect Dis.

[22] Katouli M, Jaafari A, Farhoudi-Moghaddam A, Ketabi G (1990). Aetiological studies of diarrhoeal diseases in infants and young children in Iran. J Trop Med Hyg.

[23] Katouli M, Pachenary A, Jaafari A, Moghaddam AAF, Dehaghi NH, Mirfattah ZA (1989). The role of *Shigella* spp. in childhood diarrhoea in Iran and their antibiotic resistance. Scand J Infect Dis.

[24] Yamashiro T, Nakasone N, Higa N, Iwanaga M, Insisiengmay S, Phounane T (1998). Etiological study of diarrheal patients in Vientiane, Lao People’s Democratic Republic. J Clin Microbiol.

[25] Gascon J, Vargas M, Schellenberg D, Urassa H, Casals C, Kahigwa E (2000). Diarrhea in children under 5 years of age from Ifakara, Tanzania: a case-control study. J Clin Microbiol.

[26] Ansari S, Sherchand J, Parajuli K, Mishra S, Dahal R, Shrestha S (2012). Bacterial etiology of acute diarrhea in children under five years of age. J Nepal Health Res Counc..

[27] Farahani NN, Jazi FM, Nikmanesh B, Asadolahi P, Kalani BS, Amirmozafari N (2018). Prevalence and antibiotic susceptibility patterns of *Salmonella* and *Shigella s*pecies isolated from pediatric diarrhea in Tehran. Arch Pediatr.

[28] Talebreza A, Memariani M, Memariani H, Shirazi MH, Shamsabad PE, Bakhtiari M (2016). Prevalence and antibiotic susceptibility of Shigella species isolated from pediatric patients in Tehran. Arch Pediatr Infect Dis.

